# Identifying Crohn’s disease signal from variome analysis

**DOI:** 10.1186/s13073-019-0670-6

**Published:** 2019-09-30

**Authors:** Yanran Wang, Maximilian Miller, Yuri Astrakhan, Britt-Sabina Petersen, Stefan Schreiber, Andre Franke, Yana Bromberg

**Affiliations:** 1 Department of Biochemistry and Microbiology, Rutgers University, New Brunswick, NJ USA; 2 Present address: Elastic NV, Jersey City, NJ USA; 3 Institute of Clinical Molecular Biology, Christian-Albrechts-University of Kiel, Kiel, Germany; 4 Department of Internal Medicine I, University Hospital Schleswig-Holstein, Kiel, Germany; 5 Department of Genetics, Rutgers University, Piscataway, NJ USA; 6 Technical University of Munich Institute for Advanced Study, (TUM-IAS), Lichtenbergstr. 2a, 85748 Garching, Germany

## Abstract

**Background:**

After years of concentrated research efforts, the exact cause of Crohn’s disease (CD) remains unknown. Its accurate diagnosis, however, helps in management and preventing the onset of disease. Genome-wide association studies have identified 241 CD loci, but these carry small log odds ratios and are thus diagnostically uninformative.

**Methods:**

Here, we describe a machine learning method—AVA,Dx (Analysis of Variation for Association with Disease)—that uses exonic variants from whole exome or genome sequencing data to extract CD signal and predict CD status. Using the person-specific coding variation in genes from a panel of only 111 individuals, we built disease-prediction models informative of previously undiscovered disease genes. By additionally accounting for batch effects, we were able to accurately predict CD status for thousands of previously unseen individuals from other panels.

**Results:**

AVA,Dx highlighted known CD genes including *NOD2* and new potential CD genes. AVA,Dx identified 16% (at strict cutoff) of CD patients at 99% precision and 58% of the patients (at default cutoff) with 82% precision in over 3000 individuals from separately sequenced panels.

**Conclusions:**

Larger training panels and additional features, including other types of genetic variants and environmental factors, e.g., human-associated microbiota, may improve model performance. However, the results presented here already position AVA,Dx as both an effective method for revealing pathogenesis pathways and as a CD risk analysis tool, which can improve clinical diagnostic time and accuracy. Links to the AVA,Dx Docker image and the BitBucket source code are at https://bromberglab.org/project/avadx/.

**Electronic supplementary material:**

The online version of this article (10.1186/s13073-019-0670-6) contains supplementary material, which is available to authorized users.

## Background

Crohn’s disease (CD) is a chronic inflammatory bowel disease (IBD) of the gastrointestinal tract with an incidence up to 29.3 cases per 100,000 person-years [1], affecting as many as 780,000 people in the USA alone [2]. Chronic inflammation, a hallmark of CD, may occur in any part of the gastrointestinal tract and may in some cases also manifest extraintestinally [3]. A combination of genetic, microbiome, and environmental factors is involved in disease etiology [4, 5]. Genome-wide association studies (GWAS) contribute to the understanding of the genetic architecture of CD and have, so far, identified 241 significantly associated loci [6]. These findings elucidate the underlying molecular disease pathways, contributing to the understanding of the fundamental biology behind CD pathogenesis. GWAS results highlight the roles of the endoplasmic reticulum stress [7], barrier integrity [5], innate immunity [8], autophagy [9], cytokine production [10], lymphocyte activation [10], the response to bacteria, and specifically the role of the JAK-STAT-pathway [10]. However, with few exceptions, individual risk loci confer only a modest effect on disease susceptibility. Altogether, the known loci explain approximately 13% of disease incidence [11]. Thus, definitive CD diagnosis still requires a combination of endoscopic, histological, radiological, and/or biochemical investigations [12]. Several serologic markers, primarily anti-*Saccharomyces cerevisiae* antibody (ASCA) and perinuclear anti-neutrophilic cytoplasmic antibody (pANCA), have recently been suggested to be clinically useful for diagnosis [13, 14]. However, these markers are not accurate enough to precisely diagnose CD on their own and are, therefore, used to supplement conventional tests. Moreover, for up to 14% of IBD patients, the diagnosis changes during the course of disease [15], suggesting that some are erroneously diagnosed and may even be treated for the wrong disease.

The predictive value of genetic testing for the disease-associated variants is controversial since the identified mutations generally exhibit weak correlation and do not identify causative patterns. Still, computational predictions, based on 30 GWAS CD loci, have attained a fairly high accuracy with an area under the receiver operating characteristic curve (ROC AUC) of 0.71 on simulated data that can be further improved to 0.74 by incorporating family history [16]. In another study, a logistic regression model attainted even better reported predictive performance (ROC AUC = 0.86) by training on 573 GWAS loci in over 13,000 individuals [17]. Note that when this model was applied our panel of patients and controls performance was worse than expected (ROC AUC = 0.63 for CD-train panel), possibly because our exome sequencing did not cover the majority of the necessary loci.

While CD GWAS-based models may have high predictive ability, they require large panel sizes for identification of the (necessarily common) significant loci. Whole exome (WES) or genome (WGS) sequencing can provide an alternative, pathogenesis pathway-oriented, perspective, as many of the rare or private single nucleotide human exome variants (SNVs) are functionally significant [18].

Here, we show that health status predictions based on functional effects of all individual-specific non-synonymous variants can be used to discriminate between CD patients and healthy individuals (HC). Using the *Pascal* method [19], we identified the genes most likely to be CD-relevant on the basis of GWAS summary statistics. For each gene in this set, we then computed its function score, per individual in our panel, on the basis of predicted functional effects of all its variants. The support vector machine (SVM) trained to recognize people as CD or HC attained a ROC AUC of 0.70—a performance similar to findings reported above. Note that model performance was far worse when our scoring function for these genes only accounted for the number of variants per gene (variant burden), rather than their effects on molecular functionality. These results suggest that changes to molecular functions of affected genes are more representative of disease-associated pathway deficiencies than the number of variants per pathway alone.

We further used computational feature selection (FS) techniques to directly identify CD-relevant genes from our exome data, instead of using predetermined (*Pascal*) genes. This approach improved model performance (ROC AUC = 0.74). We termed the combination of our gene selection and model training approach AVA,Dx—Analysis of Variation for Association with Disease X, i.e., we believe that AVA,Dx is generic enough to be applied to other diseases. This approach did not incorporate any prior knowledge of CD biology, and our selected genes were not significantly overlapping with any of the previously identified sets of genes. These findings suggest that AVA,Dx may reveal previously unseen Crohn’s disease pathogenesis pathways.

To test the true predictive performance of our model, we optimized batch (and sequencing platform) effect removal algorithms specifically to our data type. Remarkably, our method was able to make similarly accurate predictions (CD-test panel ROC/PR AUC = 0.69/0.92 and WTCCC-GTEx combined panel ROC/PR AUC = 0.76/0.94) for individuals from vastly different panels.

Finally, we note that our approach has so far required only a very small set of people to draw conclusions. Moreover, while we only included the exonic information from WES, there is a lot of regulatory information in this data as well. Larger training panels and additional features, including regulatory variants and, potentially, environmental factors (e.g., human-associated microbiota), are expected to improve model performance. However, current results already position AVA,Dx as both an effective method for highlighting pathogenesis pathways and as a simple CD risk analysis tool, which can improve clinical diagnostic time and accuracy.

## Methods

### Individuals in the study

Four panels of individuals were used in this study (Additional file 1): CD-train (https://genomeinterpretation.org/content/4-crohns-exomes), CD-test (https://genomeinterpretation.org/content/crohns-disease-2013), WTCCC panel (EGAD00001000401, European Genome-Phenome Archive), and GTEx panel (phs000424, Genotype-Tissue Expression Project). All samples of CD-train and CD-test and information on their corresponding phenotypes were obtained from the PopGen Biobank (Schleswig-Holstein, Germany).

The CD-train panel included 64 unrelated CDs and 47 unrelated HCs. To avoid overfitting of models by family, we additionally checked for relationships in CD-train panel using genetic data and found S076&S111 and S087&S110 to be related. These two pairs were treated as being in the same family in all cross-validations in the study, i.e., we performed a 109-fold cross-validation as *leave-one-out* cross-validation in CD-train.

The CD-test panel included 51 CDs and 15 HCs, from 28 different pedigrees, including one monozygotic twin pair discordant for CD and eight unrelated heathy controls from a separate panel. The CD-test families were also confirmed using genetic data (Additional file 2: Section 3).

The WTCCC panel contained 2678 CD individuals. The GTEx panel contained data from 635 deceased individuals with no indication of CD, whom we consider HC. Note that the highest reported populational prevalence of CD is 0.3% [1]; thus, given the size of the GTEx panel, we expected no more than two GTEx individuals to be affected by CD.

We performed ethnicity annotation [20] of all individuals in all panels (Additional file 2: Section 2). All individuals from CD-train and CD-test were European (EUR) [21], as were most of the individuals from WTCCC and GTEx panels (Additional file 3).

We did not check whether any of the individuals in the above data sets were used in any of the earlier CD GWAS or by any of the other CD evaluation methods listed below. Thus, the performance of these outside methods may be, likely very slightly, overestimated for these panels.

### Exome sequencing and analysis

Samples from both CD-train and CD-test panels were sequenced using Illumina TruSeq Exome Enrichment Kit and the Illumina HiSeq2000 instrument. Reads were mapped to the human genome build hg19. Samples of each panel were called together using Genome Analysis Toolkit (GATK version 3.3-0) Haplotype Caller [22]. Variant calls were restricted to the TruSeq exome target. VCF data from WTCCC and GTEx panels were downloaded from European Genome-Phenome Archive and dbGaP, respectively. The VQSR (Variant Quality Score Recalibration) [22] method was employed to identify true polymorphisms in the samples rather than those due to sequencing, alignment, or data processing artifacts. For each VCF file, we ran ANNOVAR [23] to identify all variants mapping to Swiss-Prot proteins [24]. Specifically, we extracted the RefSeq mRNA identifiers from ANNOVAR output and mapped these to Swiss-Prot. Note that if a single variant mapped to more than one protein, all proteins were included into the affected set.

### Data filtering

For the training set (CD-train), we removed all variant calls on the X- and Y-chromosomes, as well as mitochondrial DNA variants. We then filtered the original VCF files with VQSR and retained only the PASS variants. Within one panel, we further cleaned the data to remove all variant loci with missing calls. Removal of these loci ensured that every individual has a confident call at every locus of the same panel. For all testing sets, we filtered variants with VQSR standard and removed all variants that were not in the training set. All filtering was done using VCFtools [25] and BCFtools [26, 27] (see details in Additional file 2: Section 1).

### Gene scoring

We first checked the Swiss-Prot [24] protein sequence for correspondence, i.e., we looked for the variant-defined wild-type residue to exist in the variant sequence position. If the position contained the mutant amino acid instead, we assumed allele disagreement between reference databases RefSeq and Swiss-Prot. For these variants, we chose the RefSeq sequence to be correct and replaced the amino acid in the Swiss-Prot sequence to correspond to RefSeq. We then computed the raw SNAP [28, 29] score for each variant, ranged from − 100 to 100, where any score less than or equal to zero is classified as neutral, i.e., no protein function change, and non-neutral otherwise. Note that we used SNAP “as-is,” i.e., no changes were made to the method.

An individual *variant score* (*v_score*) was assigned as follows, for:

1. Non-synonymous variants
  - SNAP score ≥ 0 (effect): *v_score* = 0.06 + (SNAP score/100) × 0.94
  - SNAP < 0 (neutral): *v_score* = 0.055
2. Synonymous variant, *v_score* = 0.05
3. InDel variants, *v_score* = 1
4. Erroneously mapped variants and variants in 11 genes that could not be handled by SNAP (genes > 6000 amino acids), *v_score* = 0.055

SNAP score of non-neutral (effect) variant was standardized to fit a 0 to 1 range (0 and 1 represented no mutation and knockout of function, respectively) and to account for overarching effect/no effect classification. No effect for non-synonymous variants was similar to having a synonymous variant—a fixed small score (0.055). Indels were fixed to large scores (1)—this scoring was not optimized, but rather heuristically chosen to represent likely functional effects of variants. Individual *v_scores* of heterozygous variants were multiplied by 0.25 (in Eq. 1 *het* = 0.25 for heterozygous and *het* = 1 for homozygous variants) to approximate the effects of heterozygosity.

For every gene in every individual, we computed a gene functional deficit score (*gene_score*) as a sum over all gene-specific *v_scores* (Eq. 1). Note that gene scores computed in this fashion are zero only for genes that have no variants at all. However, further comparison between gene scores for different genes is not possible, as the score is highly dependent on gene length and overall tolerance for variability, e.g., longer genes with more variable regions will tend to score higher while remaining relatively functional biochemically.

Thus, for each gene, *g*, the overall variant burden score of all *N*_*g*_ variants was:

1 $$ gene\_ score(g)={\sum}_i^{N_g}{het}_i\times v\_{score}_i $$

In our representation, thus, every individual exome can thus be viewed as a vector of individual gene scores with an associated binary disease class (status: CD vs. HC). All exome vectors of one panel of individuals are of the same length, i.e., genes that are not affected by any variants in a particular individual are assigned a zero score. Genes with no variants in any individual in a panel were removed from consideration. We also removed genes that have consistent non-zero scores within one panel and genes that were only mutated in one individual (i.e., had only one non-zero score) in the entire panel. Besides *gene_score*, we tested the performance of another four gene scoring schemes (Additional file 2: Section 4) as well.

### Reference candidate gene set extraction

We extracted CD-related genes by five different approaches (see details in Additional file 2: Section 5): (1) genes selected via natural language processing of abstracts indexed by PubMed with medical subject heading, MeSH, terms relating to Crohn’s disease (*MeSH set*, 2471 genes); (2) genes in linkage disequilibrium (LD) with the known GWAS-established CD loci [10] (*unranked-GWAS set*, 1286 genes); (3) a set of all proteins annotated as Crohn’s disease related (Disease feature) in Swiss-Prot [24]. (*SP set*, 22 genes); (4) *Pascal* [19] ranked list of CD-related genes from CD GWAS summary statistics, 393 genes with a Benjamini and Hochberg corrected *p* val < 0.05 (*PascalGWAS set*, 312 genes from *PascalGWAS set* were in CD-train); (5) 50 genes associated with very early onset (VEO) IBD reported by Uhlig et al. [30] (*VEO set*, only 36 genes of the *VEO set* contained variants in at least one individual in CD-train). Additionally, all genes where at least one individual in CD-train had at least one variant were termed *ALL set*. In text, a subscript number following the set name indicated the gene number of top-ranked genes from this set used to build models. For example, *PascalGWAS*_*100*_ indicated building a model using top-ranked 100 genes from *PascalGWAS set*.

### Feature selection (FS) candidate gene set extraction

We performed the following gene set selections from the CD-train panel:

1. Collected genes where at least 3 CDs and no HCs had non-zero *gene_scores* (*disease set*, abbreviated as *DIS set*)
2. Compared the distribution of *gene_scores* for CDs vs. HCs using the *t test* (*TT5 set*) and *Kolmogorov-Smirnov test* (*KS5 set*) and took the genes that were differently (*p* < 0.05, no correction for multiple testing) distributed in CDs and HCs
3. Applied DKMcost feature selection [31] (from R CORElearn package [32]) and ranked genes by their merit

In order to avoid overfitting, we applied the above FS techniques (*DIS*, *KS5*, *TT5*, and *DKMcost*) in a *leave one out* fashion, iteratively in each fold of cross-validation (see the “CD models” section—*training cross-validation*). Thus, we had built multiple AVA,Dx models of CD-train-based with different gene sets each using the same FS technique, so that no model was trained and tested on the same samples. As a “sanity check,” we collected genes as described in method (1), *DIS set*, above from the entire CD-train panel (overfitting, *DISO set*), and trained *DISO* gene models in a leave-one-out fashion. Subscript numbers, e.g., *KS5*_*r100*_ or *DKMcost*_*125*_, meant the random (_*r*_) or top ranked (_125_), respectively, number of genes used in building the model as described in the “Reference candidate gene set extraction” section*.* When all genes from the *FS set* were used to build a model, the gene name was followed by a subscript *max* (e.g., *KS5*_*max*_).

### Computing gene set overlap

As described above, the number of FS candidate gene sets for one panel and one extraction technique was equal to the number of unrelated individuals in that panel, e.g., there were 109 different *KS5 sets* in a 109-fold cross-validation on CD-train data. For calculation of overlap between any *KS5 set* and a gene set with fixed genes, e.g., *MeSH set*, we computed the overlap and the significance (hypergeometric distribution test against a background of the corresponding variant-affected genes) for all 109 *KS5 sets* and recorded the mean. For calculation of overlap between two non-fixed gene sets, e.g., between *KS5* and *DKMcost sets*, we computed the overlap and significance when the same test individual was held-out, and recorded the mean.

### Finding gene networks

We used the ConsensusPath database [33–35] to identify the enrichment in alterations of the known molecular pathways in the selected CD-train genes. *ALL set* of CD-train was used as the background list. *KS5*_*max*_ and *DKMcost*_*125*_ selected from the entire CD-train panel, as well as *PascalGWAS*_*175*_ genes, were used as input for the pathway enrichment analysis (pathways with a *q* val < 0.1 in Additional file 4). Induced network analysis from ConsensusPath database using FS genes as starting points was used to detect additional potentially CD-associated genes.

### CD models

#### Training: model building and cross-validation

We built CD models using leave-one-out cross-validation on the CD-train panel. Note that individuals of the pair S087 & S110 and the pair S076 & S111 are more genetically similar than others and potentially related (Additional file 5), so we left the members of each of these pairs out simultaneously in our leave-one-out cross-validation, i.e., we performed a 109-fold cross-validation on the CD-train data of 111 individuals. For each model, to make the classes of the training set balanced for CD vs. HC individuals, we bootstrapped the individual samples of the minor class (resampling with replacement) to create new training samples in a balanced manner. All models used the Support Vector Machine (SVM), algorithm in R’s e1071 package [36]. Note that changing the learning method, i.e., replacing SVM with Naïve Bayes, neural networks, etc. or adjusting method parameters, could potentially produce better results. However, as the goal of this experiment was to evaluate the CD relevance of the selected gene sets, we did not optimize algorithm performance. For evaluating the performance of the different gene selection methods, we:

1. Randomly sampled with replacement different numbers (10, 25–300, in steps of 25) of genes, 100 times from each cross-validation fold gene set. The gene number was recorded as a subscript following the gene set name as described in “Reference candidate gene set extraction” and “Feature selection (FS) candidate gene set extraction” sections. For example, for 100-gene *KS5 set* (*KS5*_*r100*_) in CD-train, this meant that we trained 10,900 models—100 random gene sets for each cross-validation fold. Note that when the gene set did not have enough genes for sampling, we used the entire set to build models—one model per fold (*max* subscript following the gene set name, e.g., *KS5*_*max*_). For example, if the *KS5 set* had 113 genes, models requiring more than that used the whole KS5 (*KS5*_*max*_) set in every model training iteration. Note that for all models built using a fixed set of genes, the only source of difference in model performance is the differential resampling of the training individuals of the minor class.
2. We took the top-ranked 10 or 25 to 300 (in steps of 25) genes and performed cross-validation with the same top-ranked genes for each fold of cross-validation: e.g., *DKMcost*_*50*_ means we trained 10,900 models—top-ranked 50 *DKMcost* genes for each testing fold. Here as above, the only source of the difference in model performance is the differential resampling of the training individuals of the minor class.

For each gene set, we computed the various model performance metrics, including AUC of the precision-recall (PR) and ROC curves (R package PRROC [37], Eq. 2, where TP = true positives, correctly identified individuals with CD; FP = false positives, healthy individuals misclassified as having CD; TN = true negatives, correctly identified healthy individuals; and FN = individuals with CD misidentified as being healthy).

$$ \mathrm{Precision}\ \left(\mathrm{positive}\ \mathrm{predictive}\ \mathrm{value}\right)=\frac{\mathrm{TP}}{\mathrm{TP}+\mathrm{FP}} $$

2 $$ \mathrm{Recall}\ \left(\mathrm{sensitivity},\mathrm{true}\ \mathrm{positive}\ \mathrm{rate}\right)=\frac{\mathrm{TP}}{\mathrm{TP}+\mathrm{FN}} $$

$$ \mathrm{False}\ \mathrm{positive}\ \mathrm{rate}=\frac{\mathrm{FP}}{\mathrm{FP}+\mathrm{TN}} $$

Class labels for CD and HC were set at 100 and − 100, i.e., more negative scores indicate likely healthy individuals and more positive scores indicate likely CD patients. We obtained ROC and PR curves by varying the threshold for classifying an individual as CD-affected or healthy from − 100 (most healthy) to 100 (most CD).

To further test if the performance was achieved by chance, we did permutation test by shuffling the labels of every training fold in the cross-validation 1000 times. Null distributions of ROC and PR AUCs were based on these 1000 results. The permutation *p* values for the “real” cross-validation AUCs were obtained empirically by counting the number of larger permuted AUCs (divided by 1000).

#### GWAS-based predictions

We compared the prediction performance of AVA,Dx to two GWAS-based CD evaluation methods (see details in Additional file 2: Section 6). Briefly, (1) we calculated the polygenic risk score (PRS) for all individuals, using the log odds ratios of the 230 CD-relevant EUR loci [38], and (2) we obtained a CD logistic regression model from a previous study [17].

#### Eliminating inter-panel batch effects of sequencing

To remove the batch effect, we applied the ComBat method [39] (from R package sva [40]). ComBat is an empirical Bayes framework for adjusting (originally) gene expression data for batch effects. Here, we applied ComBat to the *gene_scores*, which represent the gene functional changes instead. To simulate the “real-world situation” of predicting disease, ComBat was applied individually to each person from the test sets vs. the entire CD-train panel. Note that since in this case the unknown batch has only one sample, only the means, and not the variance, of the *gene_scores* were adjusted. Also, note that *gene_scores* of the testing individual were adjusted against the entire CD-train panel *regardless of the class label*, i.e., we did not use the class label of the testing individual in the batch effect removal process (details in Additional file 2: Section 7).

#### Prediction models

We used the entire unadjusted CD-train panel to select *DKMcost*_*125*_ *genes* as fixed features for our final model*.* We then tested the predictive ability of our model by predicting the health status of 62 individuals from the CD-test panel, 2488 from the WTCCC panel, and 544 from the GTEx panel (all EUR, duplicated individuals removed, see details in Additional file 2: Section 3). We performed the prediction and evaluation for the non-EURs as well (Additional file 2: Section 8). Specifically, for each exome, (1) the batch effect was removed as described above, that is, *gene_scores* of the testing individual and the CD-train panel were adjusted towards the same mean regardless of the class label; (2) the CD-train panel was resampled to create 500 individuals of each HC and CD class; and (3) a model was trained on these 1000 individuals to make a prediction for the test individual. Note that the test individuals were never seen by the corresponding models.

#### Choosing the default prediction cutoff

We once more built models of CD-train in cross-validation as described above, but this time we also resampled individuals in each fold of training to create 500 individuals of the CD and HC classes. We used the originally selected *DKMcost*_*125*_ gene sets for each of the 109 models and tested on the left-out individuals. We computed the means of the prediction scores of the CD-train set CD and HC individuals, choosing the mean of these two means as the default cutoff. As the cutoff varied with different resampling and training rounds, we conducted this process 1000 times and chose the most common cutoff value for subsequent predictions.

When predictions of all individuals were made, we evaluated the performance by computing the MCC (Matthews correlation coefficient, Eq. 3), and both CD and HC precision, recall, and *F*_1_ score (Eq. 4) at different cutoffs. We also computed the AUC for the ROC and the PR curves for both CD and HC classes. Note that a baseline ROC AUC is 0.5. The baseline PR AUCs here are 0.58, 0.79, 0.82, for CD-train, CD-test, and the WTCCC-GTEx (only EUR) panels, respectively, i.e., the number of positive samples (here CD) divided by the number of total samples. The significance of the prediction result was also evaluated empirically by a 1000-time permutation test as described in *cross-validation.*

3 $$ \mathrm{MCC}=\frac{\mathrm{TP}\times \mathrm{TN}-\mathrm{FP}\times \mathrm{FN}}{\sqrt{\left(\mathrm{TP}+\mathrm{FP}\right)\left(\mathrm{TP}+\mathrm{FN}\right)\left(\mathrm{TN}+\mathrm{FP}\right)\left(\mathrm{TN}+\mathrm{FN}\right)}} $$

4 $$ {F}_1=\frac{2\times \mathrm{TP}}{2\times \mathrm{TP}+\mathrm{FP}+\mathrm{FN}} $$

## Results

### AVA,Dx pipeline

**Fig. 1:**
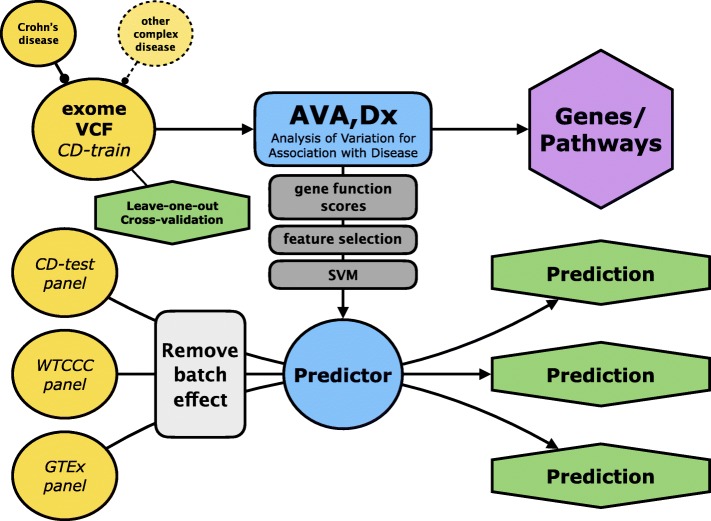
AVA,Dx pipeline flowchart. A simplified pipeline of AVA,Dx (Analysis of Variation for Association with Disease). Input data is in variant call format (VCF) file from whole exome sequencing. Different versions of *gene_scores* are calculated as described in the “Methods” section. Models for prediction of Crohn’s disease status are evaluated by cross-validation. Best *gene_score* scheme, FS algorithm, and, finally, SVM model are selected on the basis of performance in cross-validation. These are further used for prediction of unknown individuals

We constructed the AVA,Dx pipeline as outlined in Fig. 1 and the “Methods” section. Briefly, we performed predictions of functional effects of variants and converted the latter into per-gene scores (*gene_score*) of individual-specific functionality. To build predictive models, we (1) considered externally determined disease genes (e.g., GWAS and literature-identified genes, “Methods” section and Additional file 2: Section 5) and (2) extracted gene sets via computational feature selection (FS), which identified previously unreported CD-related genes (“Methods” section). SVM [41] models using these gene sets were trained in leave-one-out cross-validation on the training panel of individuals (CD-train; all individuals of 1000 Genomes Project [42] EUR, European descent, designation). We further applied permutation testing for each model to calculate the empirical *p* values, which show that our prediction performance was significantly non-random (“Methods” section).

We tested four different gene scoring schemes in addition to our default *gene_score* (Additional file 2: Section 4). Our *gene_score* outperformed all other scoring schemes in testing (Additional file 2: Figure S1), highlighting the importance of using the severity of functional effects of variants in evaluating disease genetics. We also looked for variants in the CD-train panel that may be associated with the CD phenotype (Fisher’s exact test with false discovery rate correction). However, due to panel size and, possibly, sequencing/data filtering issues, no significant associations were found (Additional file 2: Table S1). We thus used *gene_score* in all further analyses.

For prediction of individuals from different panels, we only considered the variants that were also present in CD-train individuals and calculated the *gene_scores*. The ComBat algorithm [39] was used for batch effect removal of the *gene_score* differences between the training set and each test individual. The CD-train SVM model was further tested on the individuals from CD-test, WTCCC (all CD), and GTEx panels (all healthy controls, HC; “Methods” section).

### Pascal-ranked CD GWAS genes differentiate CDs from HCs

**Fig. 2:**
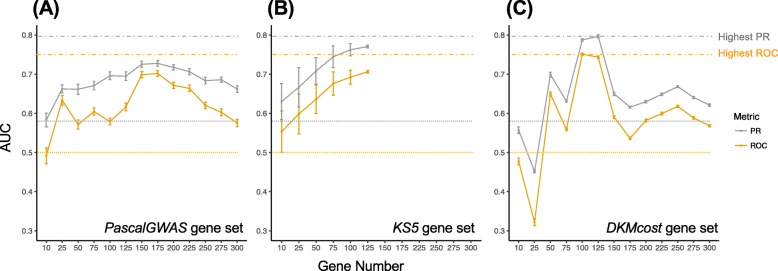
*DKMcost* genes outperform *PascalGWAS* and *KS5* sets in leave-one-out cross-validation on the CD-train panel. The *x*-axis is the number of genes/features used in each model. The *y*-axis is the AUC for precision/recall (PR, gray) and ROC (yellow) curves. At each point on the graph, the SVM models were trained using **a** top-ranked genes from the *PascalGWAS* genes, **b** randomly selected *KS5* genes; note that at most 127 genes were in *KS5*, i.e., were below the Kolmogorov-Smirnov *p* value of 0.05 (“Methods” section), **c** top-ranked *DKMcost* genes. Error bars are standard deviations over 100 iterations of model training. Note that for each point on the *x*-axis in **a** and **c** the genes used in each of these training iterations were the same, but the resampling of individuals was different. Dotted lines indicate baseline performance of ROC AUC of 0.5 (yellow) and PR AUC of 0.58 (gray, 64 CDs out of 111 total number of individuals). Dashed lines indicate the highest performance achieved through all gene sets

The *Pascal* [19] top-ranked CD GWAS genes (*PascalGWAS set*, “Methods” section), scored for functional effects (Eq. 1), were used to build SVM models on the CD-train set, as described above. These models achieved much better performance than models using random genes from other external (known CD) gene sets including unranked *GWAS*, *MeSH*, *Swiss-Prot*, and *VEO* gene sets (Additional file 2: Figure S2). Our models achieved the highest ROC AUC of 0.70 (PR AUC = 0.73) using 175 Pascal top-ranked genes (*PascalGWAS*_*175*_ genes, Fig. 2a). By further permuting (“Methods” section) the CD/HC labels in cross-validation, we showed that the performance of our models is significantly non-random (ROC/PR permutation *p* val = 0.001/0.011). Note that here and in all fixed gene sets, the only source of the difference in model performance is the differential resampling of the training individuals of the minor class (“Methods” section).

### Feature selected (FS) genes outperform Pascal genes in differentiating CDs from HCs

We further evaluated the performance of the computationally extracted FS genes (*DIS/DISO*, *KS5*, *TT5* and *DKMcost sets*; “Methods” section). Trivially, the best performance (ROC/PR AUC = 1/1) was achieved by the *DISO* (*Disease Overfitted*) *sets* of more than 100 genes, defined as genes that were not affected in any of the CD-train HCs (“Methods” section).

Both the *KS5*_*max*_ (all genes in *KS5 set*, ROC/PR AUC = 0.71/0.77, permutation *p* val = 0.033/0.022) and the *DKMcost*_*125*_ (top-ranked 125 genes from *DKMcost set*, ROC/PR AUC = 0.75/0.80, permutation *p* val = 0.014/0.010) models outperformed *PascalGWAS*_*175*_ (Fig. 2b, c). For the *KS5 set*, including more genes slightly improved performance (Fig. 2b). This was not the case for *DKMcost*, whose performance had reached a peak at 125 genes before dropping off. Note, however, that the maximum number of *KS* genes was 127, suggesting that models may simply not benefit from additional genes. *TT5*_*max*_ and *DIS*_*max*_ genes also had outperformed baseline, but were not as good as *KS5*_*max*_ or *DKMcost*_*125*_ genes (Additional file 2: Figure S3). Also note that the FS sets were never overfitted to the data, as FS was performed in a leave-one-out fashion, i.e., excluding the testing individual. Thus, our results suggest that FS selected genes can differentiate CDs from HCs in our data, particularly using rare variant signal that is not available to the common variant-based methods.

### Feature selection identifies known and previously unreported CD genes

As described above, both *PascalGWAS*_*175*_ and FS sets (*DKMcost*_*125*_ *and KS5*_*max*_) performed well in differentiating CDs from HCs in the CD-train panel (Fig. 2). However, the FS *sets* overlapped with *PascalGWAS*_*175*_ by no more than five genes (average *p* val > 0.25, hypergeometric test, in the background of *ALL* genes, Table 1). On the other hand, the *KS5*_*max*_ set significantly overlapped with the *DKMcost*_*125*_ genes (over 57 genes; average *p* val = 6.64e−93). We also found that while the FS sets did not significantly overlap with most of the external sets (*GWAS*, *MeSH*, and *PascalGWAS*_*175*_), the external sets overlapped with each other (all with *p* val < 0.05, Table 1). Note as an exception that while *Swiss-Prot* had no overlap with *KS5*_*max*_, the overlap of the former with *DKMcost*_*125*_ was significant (NOD2 [43], MDR1 [44], and DMBT1 [45], three genes of only 18 in *Swiss-Prot*), highlighting well-known CD genes extracted computationally without prior knowledge.

**Table 1:**
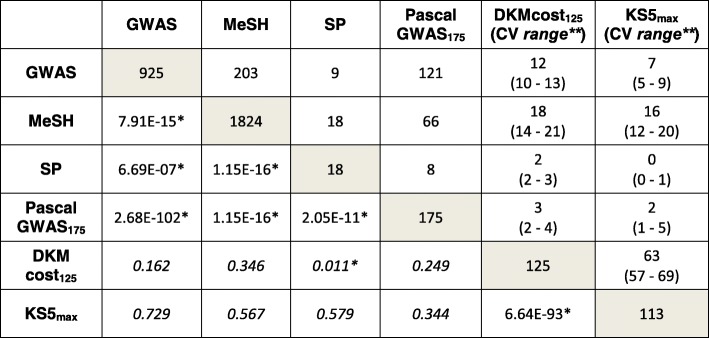
Gene set overlap summary

*Significant overlap between gene sets. The number of genes above the diagonal is the overlap between two sets. The corresponding overlap significance is below the diagonal (hypergeometric test in the background of *ALL* genes from the CD-train panel)

**There were 109 cross-validation/FS folds for each FS method (*DKMcost*_*125*_ and *KS5*_*max*_), i.e., 109 different gene sets. Here, the average fold size and range (in parenthesis) are displayed

Our FS techniques identified some known (*GWAS* and *MeSH*) genes. For example, both FS *sets* contained the CD-associated LRRK2 [46] and the uncharacterized KIAA1109 [47] genes, which also appeared in GWAS and MeSH sets. Additionally, *DKMcost*_*125*_ genes NOD2 [43], LSP1 [48, 49], and CCR6 [50] and *KS5*_*max*_ genes IL19 [8] and ATF4 [51] also appeared in *GWAS* and *MeSH*. Overall, however, few genes appeared both in the FS sets and in the experimentally derived external sets. The performance of the *KS5*_*max*_ and *DKMcost*_*125*_ models thus suggests that computational FS methods are able to identify previously unsuspected CD genes.

### CD-relevant genes interact

**Fig. 3:**
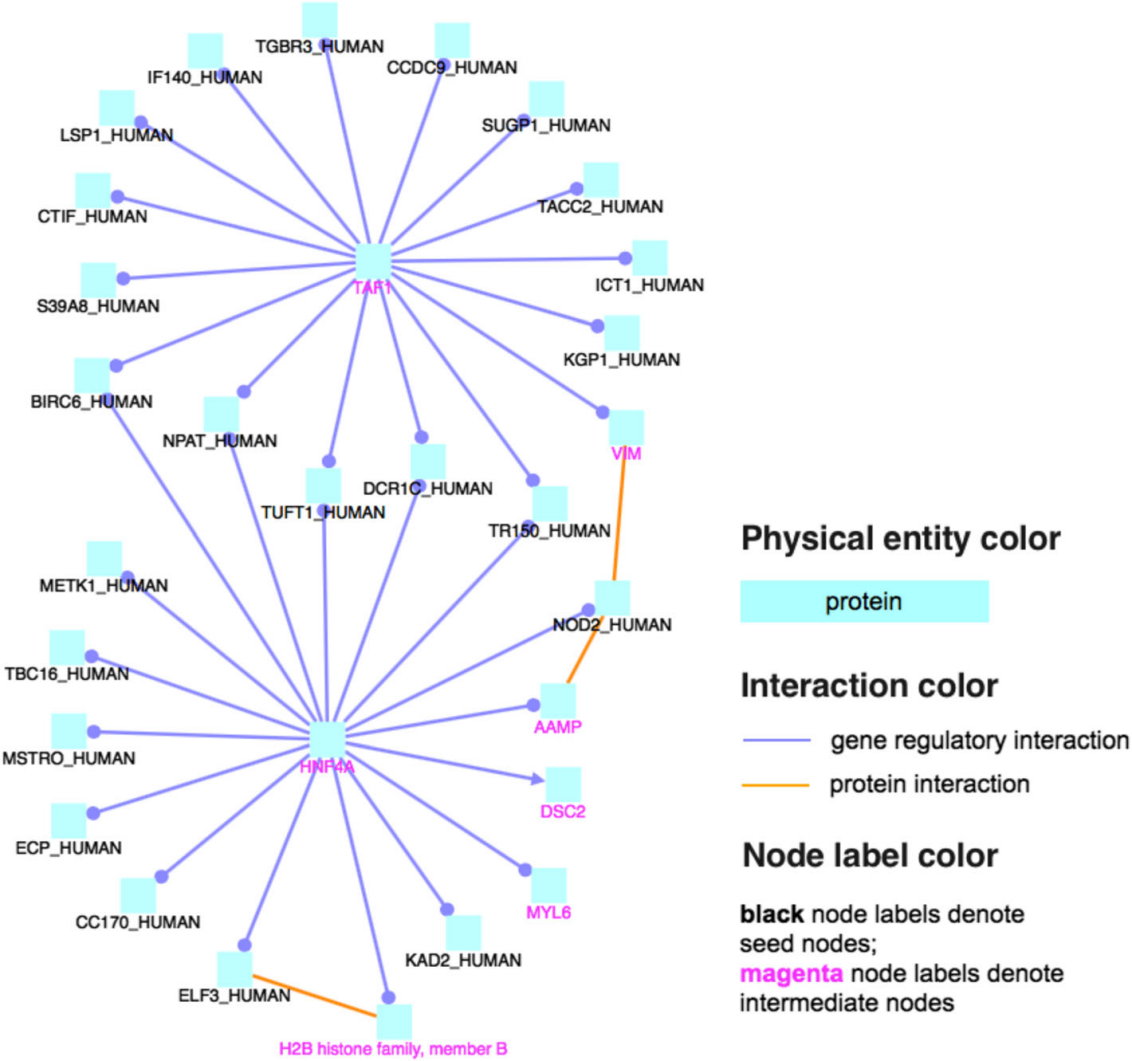
Induced network of CD-train *DKMcost*_*125*_ genes. Interconnection of *DKMcost*_*125*_ genes through protein interaction and gene regulatory interaction. Magenta nodes were genes/proteins that were not selected by FS but interact with the FS genes (black nodes) through protein or gene regulatory interactions. Transcription factors TAF1 and HNF4A regulate many *DKMcost*_*125*_ genes, indicating their potential role in CD pathogenesis

We used gene set overrepresentation analysis to check if *DKMcost*_*125*_, *KS5*_*max*_, or *PascalGWAS*_*175*_ genes are enriched in known molecular pathways. FS found several significantly enriched, likely CD-related, pathways that were not identified by *PascalGWAS*_*175*_, e.g., antimicrobial peptides, apoptosis-related pathways, cGMP effects, neutrophil degranulation, and innate immune system (Additional file 4)*.* The protein-protein interaction network of *DKMcost*_*125*_ genes (Fig. 3) suggested additional genes/proteins, which were not directly found by FS but may be relevant to CD, e.g., the TAF1 and the HNF4A transcription factors regulate many *DKMcost*_*125*_ genes, including the infamous NOD2. HNF4A was annotated as CD-associated in previous studies [52]. TAF1, on the other hand, needs further evaluation, but preliminary analysis shows that it contains a bromodomain, which may be critical in inflammation in general and bowel inflammation [53–55].

### At high scoring thresholds, AVA,Dx precisely identifies people affected by Crohn’s disease

#### Cutoff selection

**Fig. 4:**
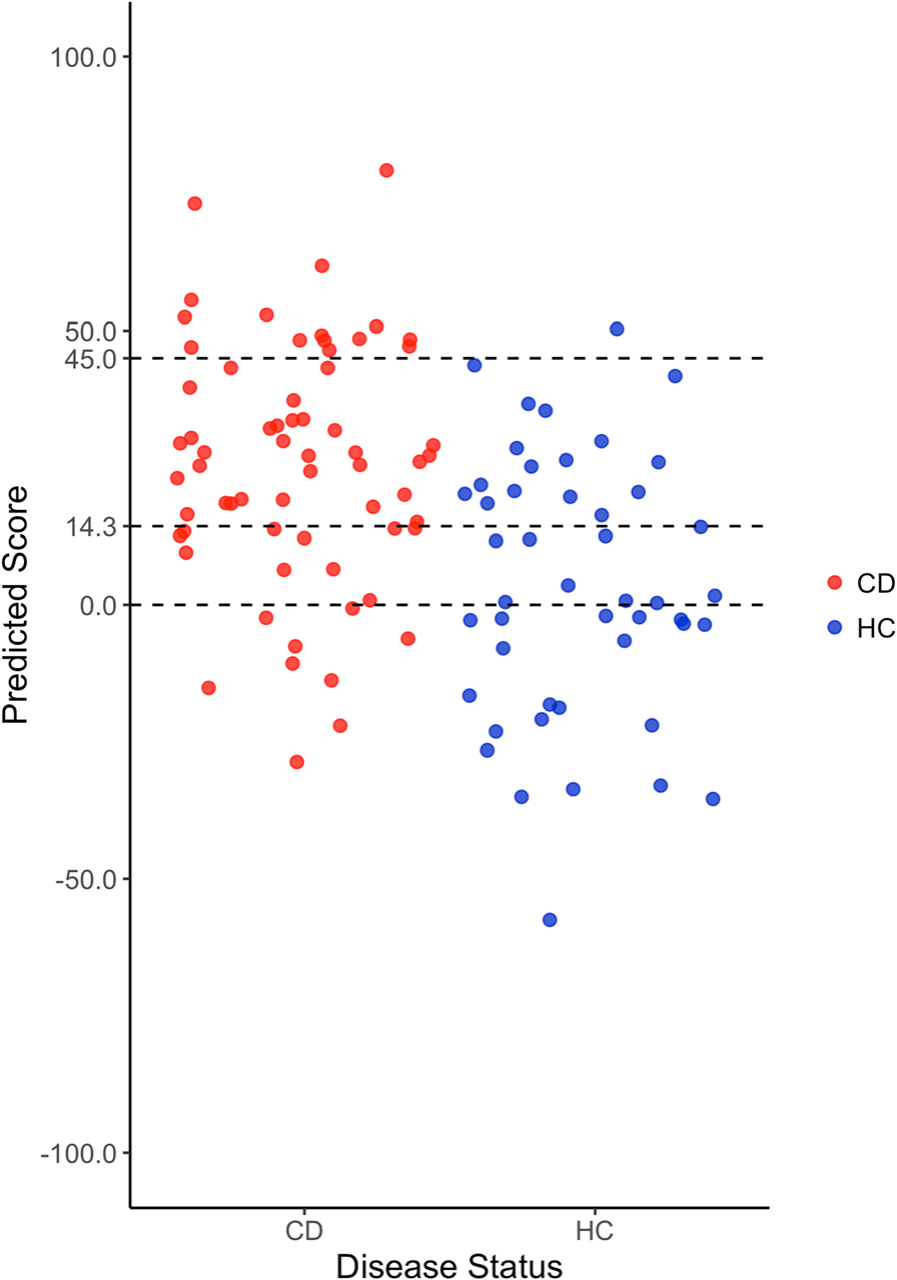
Prediction of CD-train individuals using the *DKMcost*_*125*_ model in leave-one-out cross-validation. Each dot represents one person in the panel, colored according to health status (red = CD, blue = HC). Dots are jittered along the *x*-axis for better visualization. Dashed lines are the three cutoffs used for different levels of prediction stringency in calling an individual a CD patient. The predicted scores of CD and HC individuals were significantly different (Kolmogorov-Smirnov test *p* value = 0.0002226)

To select the cutoff in AVA,Dx score for calling an individual healthy or CD-affected, we plotted the prediction scores for each individual from CD-train in cross-validation and selected a cutoff that best differentiated CDs from HCs (Fig. 4, Additional file 2). We chose three cutoffs as follows: (1) The default cutoff was set at 14.3 (“Methods” section), where we had balanced precision and recall for both CDs and HCs in our set (47 of 64 CD patients were correctly identified, as were 28 of 47 healthy controls; 71% precision, 73% recall, Matthews correlation coefficient (MCC) = 0.33). (2) To precisely identify CD, we set a stricter cutoff at 45, where 94% of the individuals above the cutoff were sick (27% recall). (3) On the other hand, to identify as many CD patients as possible, we set a cutoff at 0 where 89% of the CD patients were identified (70% precision). This tradeoff between precision and recall of predictions across thresholds suggest using the individual AVA,Dx scores to estimate the reliability of each prediction, i.e., it is more likely that the higher scoring individuals have CD than lower scoring ones. Note, however, that the score was not evaluated and should not be used as an indicator of disease severity.

For all further analyses, we built/used *PascalGWAS*_*175*_ *and DKMcost*_*125*_ prediction models using the entire CD-train panel, as opposed to leaving one sample out for cross-validation.

#### Batch effect across panels

For AVA,Dx to be useful in diagnosis of new patients, the method has to be directly applicable to samples from different sequencing batches handled by different labs. Regardless of health status, the sequencing procedures may cover different regions on the genome/exome and potentially result in different numbers of variants even for the covered regions (Additional file 2: Section 7). This “batch effect” is a likely result of the use of diverse sequencing platforms and variant calling settings (Additional file 2: Tables S2 and S3). To evaluate the severity of the batch effect, we combined the *ALL gene_score* profiles of our CD-train panel with those of all other panels at our disposal (CD-test, WTCCC, and GTEx panels). We further performed principal component analysis on the combined set. Individuals clustered precisely according to batch (Additional file 2: Figure S4), suggesting that our models could not be used for prediction of individual CD status in different batches. To test new individuals, batch effects had to be removed. Moreover, to apply our method in a real-life situation, where only one individual is to be evaluated for CD at a time, the “batch” was designated as containing only one person. Thus, for each individual from the CD-test, WTCCC, and GTEx panels, we first extracted the loci covered by CD-train and then applied ComBat to adjust *gene_scores* of the entire CD-train panel and the one testing individual. We then built a new model using the adjusted CD-train panel for every testing individual—3379 models in total for the evaluation of all panels. Note that the AVA,Dx pipeline thus retrains the model to precisely fit the genomic data of every new test individual (an estimated 10 s per individual, on a 64-bit Mac iOS, with 2.9-GHz Intel Core i5 CPU and 16-GB DDR3 memory).

#### Evaluation of predictions

**Fig. 5:**
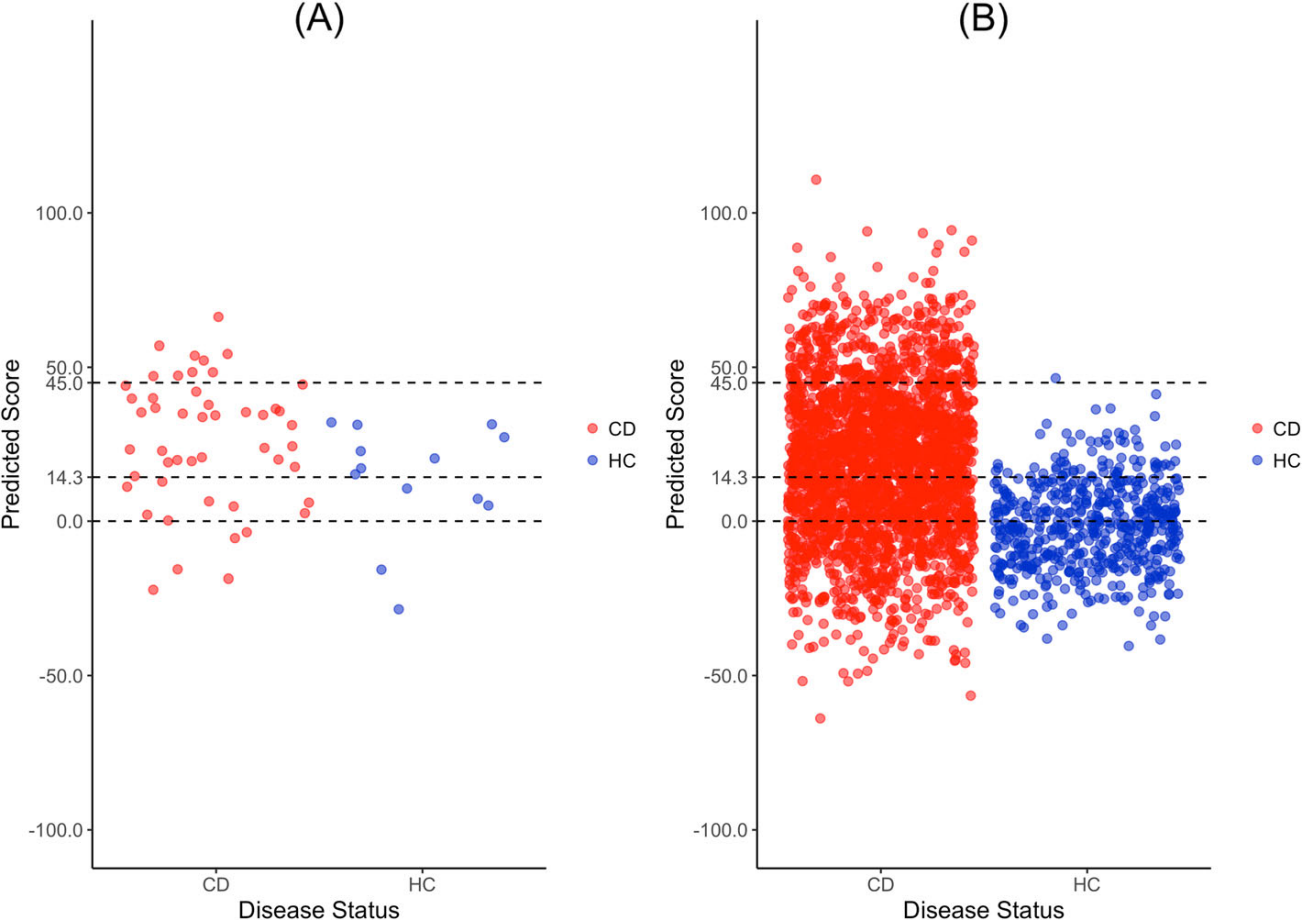
*DKMcost*_*125*_ model prediction of test set individuals. The predicted scores of all individuals from sets **a** CD-test panel and **b** WTCCC (all CD) and GTEx (all HC) panels combined. Dots are jittered along the *x*-axis for better visualization. Dashed lines are cutoffs for calling an individual a CD patient (as in Fig. 4): loose (at 0), default (at 14.3), and strict (at 45). The predicted scores of CD and HC individuals were significantly different (Kolmogorov-Smirnov test *p* values **a** = 0.007413 and **b** < 2.2e−16, respectively)

For all evaluations, as for the training set, we retained only the EUR individuals from all test sets (see performance on non-EUR individuals in Additional file 2: Section 8). The *PascalGWAS*_*175*_ model was nearly random in predicting the status of all CD-test individuals (ROC/PR AUC of 0.57/0.82, Additional file 2: Figure S5). Our *DKMcost*_*125*_ model, however, reached ROC/PR AUC = 0.69/0.92 (Fig. 5 and Table 2, permutation *p* val = 0.041/0.035, baseline PR AUC = 0.79). That is, at default cutoff, using this model, we were able to correctly identify 36 of 49 CD patients and 5 of 13 healthy controls (Table 2). Moreover, in predicting all WTCCC (CD) and GTEx (HC) individuals, the *DKMcost*_*125*_ model reached a ROC/PR AUC = 0.76/0.94 (Fig. 5 and Table 2, permutation *p* val < 0.001/0.001, baseline PR AUC = 0.82), while the *PascalGWAS*_*175*_ model failed to differentiate CD and HC (ROC/PR AUC = 0.30/0.76). Our model identified HC individuals less accurately than CD patients, but better than the baseline (*DKMcost*_*125*_ model vs. baseline PR AUCs for HC were 0.35 vs. 0.21 and 0.31 vs. 0.18 for CD-test and WTCCC-GTEx panels, respectively).

**Table 2:** *DKMcost*_*125*_ model performance on test sets

Cutoff	CD-test							WTCCC and GTEx^							
	TP	FN	TN	FP	Prec %	Rec %	MCC	TP	FN	TN	FP	Prec %	Bal. Prec %	Rec %	MCC
45	9	40	13	0	100.0	18.4	0.212	384	2104	543	1	99.7	98.8	15.4	0.176
14.3	36	13	5	8	81.8	73.5	0.107	1432	1056	476	68	95.5	82.2	57.6	0.346
0	44	5	2	11	80.0	89.8	0.067	1924	564	302	242	88.8	63.5	77.3	0.279

*TP* true positive (CDs predicted to be CD by AVA,Dx), *FN* false negative (CDs predicted to be HC), *TN* true negative (HCs predicted to be HC), *FP* false positive (HCs predicted to be CD), *Prec* precision, *Rec* recall of identifying CD patients (Eq. 2), *Bal. Prec* balanced precision, where the number of CD and HCs is standardized to represent 50% of the data, each. MCC is in Eq. 3. A more detailed performance evaluation is in Additional file 2: Tables S4 and S5

^WTCCC and GTEx panels were combined for evaluation since WTCCC contains only CD individuals and GTEx contains only HC individuals

## Discussion

After years of study on the subject and numerous promising findings, CD risk prediction on the basis of genetic information still remains a problem. We developed AVA,Dx, a machine learning method that uses individual exome data of a panel of CD patients and healthy individuals to select CD-relevant genes and, potentially, predict the health status of previously unseen individuals. We first identified the functional effects of exome SNVs and combined them to create gene scores, indicative of gene functional deficiencies*.* This approach efficiently decreases the dimensionality of data from considering all exome variants (173,013 variants) to focusing only on affected genes (13,957 genes). Additionally, FS techniques reveal new disease-related genes thus further reducing the dimensionality of data. While our method currently only considers coding variants, the path to integrating other CD-relevant types of variants, e.g., splice site and regulatory, into gene scoring is also clear.

The main idea behind AVA,Dx is that disease-causing variation is likely to be functionally detrimental to affected genes/pathway components. To evaluate whether molecular function disruption is an important indicator of gene involvement in disease, we tested a number of variant effect scoring schemes. Confirming our suspicion, we found that functional scoring was more informative than simple counting of relevant variants. Furthermore, as expected, models built using GWAS (with Pascal filtering) genes performed significantly better than random, indicating that GWAS indeed captures CD association successfully. On the other hand, our FS genes outperformed the GWAS genes, suggesting that variant functional effects are more likely to highlight causative, rather than association signals. Note, however, that AVA,Dx is not limited conceptually to the gene scoring described in this study; that is, other scores describing gene functional deficiency and including other variant types (e.g., regulatory or synonymous) and/or different genotype weighing and variant effect summation schemes can potentially be used.

Even as GWAS predictive accuracy improves, these studies are limited by large sample size requirements and use of only common SNPs. Thus, GWAS associations are often markers of disease, not causes, e.g., some disease-related genes may not be found simply because their variants are not common enough or are not covered by the SNP array. Our FS genes, on the other hand, are more informative of pathogenicity pathways, as they are selected on the basis of variation-driven gene functional changes that separate CD-affected individuals from healthy controls. Interestingly, while both FS and GWAS gene-based models both perform well, the gene sets do not have much overlap, suggesting that FS identifies previously unknown CD-related genes. We also note that for other complex or rare diseases, where GWAS data is not available or informative, AVA,Dx may work uniquely to predict health status and identify pathogenicity pathways based on even a small number of whole exome sequences.

AVA,Dx required only 111 people to build a functional model (ROC AUC = 0.75). This was less than a tenth of the ~ 13,000 individuals that were needed in an earlier study to build a GWAS logistic regression model (reported ROC AUC = 0.86). Note that using only ~ 1300 people, significantly reduced the performance of this model (reported ROC AUC = 0.60) [17]. Thus, the number of individuals in this latter type of study clearly contributed heavily to its resolution of CD risk. Interestingly, when used with our CD-train panel, the logistic regression Wei et al. model (limited to exonic variants only) was able to correctly identify nearly three quarters (46 of 64 correct) of the patients but also misidentified more than half (21 of 47 correct) of the healthy individuals. On the other hand, AVA,Dx (at the default cutoff) identified just one less CD patient correctly (45 of 64 correct), but it did so at significantly higher accuracy—mislabeling only a third of the healthy individuals (28 of 47 correct).

For the larger WTCCC and GTEx panels, where > 80% of the 573 necessary GWAS loci were covered, the logistic regression model only reached 0.59 ROC AUC (a false positive rate of 86% at default cutoff; Additional file 2: Table S4). Similarly, polygenic risk scoring [38] (PRS; all 230 CD loci, as described in the “Methods” section, were present in the WTCCC-GTEx panel) was only able to attain an ROC AUC of 0.57 (Additional file 2: Table S6). Note that the distribution of ethnic subpopulations (e.g., European American, Irish, and British) across the WTCCC and GTEx cohorts was very similar, and thus unlikely a contributing factor to performance estimates (Additional file 3). Both the logistic regression model and the PRS methods significantly underperformed AVA,Dx on this same panel (ROC AUC = 0.76).

With all of its advantages, several limitations of our method remain. First, AVA,Dx’s prediction power decreases when the exome sequences of the test panel, or the individual whose status is to be evaluated, share too few loci with the CD-train panel. There are two reasons for the difference in the covered loci—exome population of origin and sequencing quality. In case of the former, it is, arguably, not surprising that a genetic test that works for one population is not as good in, or may be not even applicable to, other populations, as is the case for other diseases (e.g., as for venous thromboembolism in African-Americans vs. Caucasians [56]). At an even finer level, different types of CD may also not be properly evaluated with a single test, as is also the case, e.g., different subtypes of breast cancer [57]. Note, however, that AVA,Dx performs similarly well for the early- and late-onset CD individuals, whose exomes were part of the training panel; the possible difference in performance could not be evaluated further as the WTCCC panel had no annotations of time of onset. The difference in sequencing quality is an even more straightforward issue—missing variant calls decrease the method power. We estimate that sharing at least 58% of the training set variants (Additional file 2: Table S3) is sufficient for prediction ROC AUC of 0.69 (i.e., CD-test); although sharing more is better (i.e., ~ 80% shared in WTCC-GTEX; ROC AUC = 0.76).

Importantly, also note that evaluating AVA,Dx performance on testing panels sequenced separately not only from the training set, but also from each other, is complicated by inter-test batch effects. That is, although we used ComBat to ameliorate the batch effects between the training panel and testing individuals, our procedure of removing batch effects for one individual at a time could not guarantee that testing panels would be non-differentiable by batch. We evaluated whether sequencing explicitly differentiates testing panels by checking their sequenced variant overlap; here, WTCCC and GTEx shared over 92% (981) of the variants in the AVA,Dx DKM gene set (of 1059 in WTCCC and 1026 in GTEx). These results suggest that sequencing differences between testing panels did not contribute significantly to the results of this study. However, more work with larger panels is necessary to evaluate the impact of batch effects on prediction performance.

Another limitation of AVA,Dx is its poor ability to recognize healthy people as healthy. The explanation for this observation is simple: our method aims to identify genetic patterns common to individuals affected by CD (a fairly well-defined panel), rather than those of healthy ones (an extremely wide set of people). Answering the latter question is akin to proving a negative—how can one be sure that the healthy people in our panel actually do not have and will never develop CD? Also note that the reliability of CD prediction is modulated by choosing a higher prediction threshold. Thus, people scoring above our strict cutoff are very likely to have CD; however, those that score just below it are termed “healthy,” which they often are not. Thus, although AVA,Dx was better than random in identifying healthy people in our panel, we do not suggest using it for these purposes.

Our method is able to use less than 5% of the people normally involved in a GWAS study to identify disease genes and to make fairly accurate CD predictions for previously unseen individuals. At high AVA,Dx scores, our method is optimized for high precision, i.e., misclassifying few healthy individuals as sick; lower AVA,Dx recall at this cutoff, i.e., failing to identify many CD patients, suggests that there are multiple CD subtypes that have yet to be clinically established. Notably, AVA,Dx is robust to differences in panels and in sequencing/filtering methods, making our approach potentially clinically useful going forward.

Furthermore, AVA,Dx-identified genes appear to be relevant to CD, as indicated by the matches of our pathways to known work, and yet significantly different from those highlighted by other methods. Thus, our method presents an *orthogonal* way for identifying disease-related genes, while avoiding the most severe research limitation—the requirement of a large study panel. This finding is in line with the higher risk expectation of causal, rather than associated, variants [58]. While a larger panel could improve performance, our results suggest that model training can also be performed using already existing panels. Note that GWAS are higher powered to stratify CD subtypes and better able to deal with ethnicity-driven differences. Most crucially, however, they can identify the disease-relevant non-coding variants. Thus, it is clear that future inclusion of the effects of regulatory, synonymous, and copy number variants is likely to improve AVA,Dx performance. Finally, we suggest that the AVA,Dx approach to model building is not limited to Crohn’s disease, but is rather applicable to a wide spectrum of genetically linked, potentially rare and complex, diseases.

## Conclusions

To summarize, we developed AVA,Dx, a tool that uses exome variant-caused gene functional changes to identify disease-related genes and make health status predictions. AVA,Dx can be used orthogonally for identifying disease-related genes. Larger panels and more comprehensive gene scoring schemes could potentially improve performance.

## Additional files

Additional file 1: Cohorts in the study. (XLSX 66 kb)

Additional file 2: Supplementary methods and results. (PDF 4720 kb)

Additional file 3: Ethnicity analysis of all individuals in the study. (XLSX 319 kb)

Additional file 4: Pathway enrichment analysis. (XLSX 14 kb)

Additional file 5: Kinship analysis of all individuals in the study. (XLSX 557 kb)

## Data Availability

CD-train and CD-test are available at the CAGI (Critical Assessment of Genome Interpretation) website (https://genomeinterpretation.org) section on Crohn’s disease challenges (CAGI-3 and CAGI-4) and are subject to the CAGI data use agreement (https://genomeinterpretation.org/data-use-agreement). Non-CAGI participants should contact the authors (Franke, A.) for data access permissions. Whole genome sequencing VCF of WTCCC panel is available in the European Genome-Phenome Archive (ID: EGAD00001000401). Whole genome sequencing VCF of GTEx panel is available in dbGaP archive (ID: phs000424).

## Abbreviations

IBD: Inflammatory bowel disease
CD: Crohn’s disease
HC: Healthy control
GWAS: Genome-wide association studies
AVA,Dx: Analysis of Variation for Association with Disease
ROC: Receiver operating characteristic
PR: Precision-recall
AUC: Area under the curve
FS: Feature selection
