# Supplementary material for: Identifying Crohn’s disease signal from variome analysis
Source: Genome Med. 2019 Sep 30;11:59. doi: 10.1186/s13073-019-0670-6 (PMC6767648; doi:10.1186/s13073-019-0670-6)
Supplement: Supplementary file 2 — Supplementary methods and results. (PDF 4720 kb) [file 13073_2019_670_MOESM2_ESM.pdf]

Additional File 2

SUPPLEMENTARY TEXT

Section 1 - Data Collection and Cleanup

Section 2 - Ethnicity annotation

Section 3 - Individual relationships

Section 4 - Comparison of different gene scoring schemes

Section 5 - Known CD genes are poorly informative of individual health status

Section 6 - Performance of the logistic regression model

Section 7 - Removing batch effect across panels

Section 8 - AVA,Dx performance for non-Europeans

Section 9 - Association tests fail to identify CD-associated SNVs

SUPPLEMENTARY FIGURES

SUPPLEMENTARY TABLES

SUPPLEMENTARY REFERENCES

SUPPLEMENTARY TEXT

Section 1 – Data Collection and Cleanup

We used four data panels: the CD-train panel (for training) for extracting the relevant genes and training the prediction model and the CD-test, WTCCC, and GTEx panels to evaluate model performance.

In the training data, CD individuals were collected at Christian-Albrechts-University of Kiel and other clinics around Germany. Healthy individuals were collected via Popgen (de-identified) and selected for not having any immunological diseases while being as old as possible to minimize the probability of them still developing such diseases. Individuals in CD-train were exclusively from Germany. Sequencing and variant calling processes were done at Christian-Albrechts-University of Kiel as described in Methods. CD-train data had a balanced sex ratio in CD and HC cohorts (28 females and 36 males in CD cohort; 20 females and 27 males in HC cohort; Fisher's Exact Test p-value=1).

For CD-train, we removed all sex chromosomes and mitochondrial variants and retained only the VQSR (1) PASS variants (GATKCommand, Version=3.3-0-g37228af: -T ApplyRecalibration -R hg19.fasta --ts_filter_level 99.0 -mode SNP). We further filtered out all variants that were missing a genotype ("./.") in even one individual; *i.e.* after filtering, all individuals in our study had genotypes for all the same variant loci. In this step, we removed 25,393 (12.8% of 198,406 in total) variants from the CD-train panel. After filtering, all loci had a QUAL score >50 (1). Further, <1% of all genotype calls had a DP < 5.

The transition/transversion ratio has increased after filtering from 2.61 to 2.69 (from 3.10 to 3.19 for the exome regions), in agreement with the accepted ratio of 2.8-3.0 for exome sequencing (2). Moreover, as it is not focused on single variant to disease association, but instead combines variant scores into a single gene score, it carries an inherent robustness to false positives. On average, 16,661 ± 125 (mean ± s.d.) exonic variants per individual sample were retained. There was no difference in variant burden between CDs and HCs (two sided Kolmogorov-Smirnov test p-val = 0.9). We used the filtered CD-train for all further analyses. For all test/evaluation individuals, we extracted only the CD-train-covered loci and did not apply any further filtering.

Section 2 - Ethnicity annotation

We annotated the ethnicity of all individuals in our study by mapping our panels to individuals from the 1000 Genomes Project (3). Specifically, we combined all individuals from our panels with the 1000 Genomes Project panel by their shared variants (98,010 SNPs). R package SNPRelate (4) was used for linkage disequilibrium (LD) pruning and principal component analysis (PCA). The centroid of each population cloud from the 1000 Genomes Project individuals was calculated and the closest centroid ethincity label (AFR, AMR, EAS, EUR, SAS; Euclidean distance) was assigned to the CD-train, CD-test, WTCCC, and GTEx individuals. Note that only the five PCs that explained > 0.1 variance were used for distance calculations. All CD-train and CD-test individuals were European (EUR), as were 2,510 (94%) WTCCC individuals and 544 (86%) GTEx individuals (Supplementary Data 3). Note that unless otherwise indicated we used only EUR individuals in each cohort for all further evaluation. We further used forensic markers from FROG-kb (https://frog.med.yale.edu/FrogKB/docFreqDownload.jsp) to annotate the ethnicities of all individuals. Specifically, we checked the 55 AISNPs markers from KiddLab in all of our cohorts. We used the formula provided by FROG-kb to calculate ethnicity, where markers detected by sequencing were used and uncovered markers were simply ignored in the formula. The exome sequencing cohorts (CD-train and CD-test) covered 4 of the markers and the whole genome sequencing cohorts (WTCCC and GTEx) covered over 50 markers. The forensic ethnicity result are listed in Additional File 3.

Section 3 - Individual relationships

To confirm individual relationships, we used kinship coefficient analysis (5) from the R package SNPRelate (4). All individuals from CD-train, CD-test, WTCCC, GTEx and 1000 Genomes Project (3) panels were combined together for relationship inference. Kinship coefficient and IBS0 (proportion of SNPs with zero identity-by-state) were recorded for every pair of individuals (Supplementary Data 3).

1. CD-train individuals S076 & S111 and S087 & S110 were related (kinship coefficient > 1/16). Thus, in the analyses throughout the study, we considered each pair as a family. Individuals from one family were always in one fold of cross-validation for both feature selection and CD model training, *i.e.* we did a 109-fold cross-validation in the *leave-one-out* process for 111 individuals of CD-train.

2. CD-test individuals made up 28 families. CD_37 & CD_63 (monozygotic twins) and CD_21 & CD_64 (mother-daughter) pairs had kinship coefficients of 0.4984 and 0.4997, respectively. This type of kinship coefficient is expected for the monozygotic twins, but not for parent-child relationships. Upon further exploration we discovered that CD_21 and CD_64 were both identified as CD patients and genetically identical, a likely recording error. We thus removed CD_21 from further consideration. We also removed the genetically identical twins from consideration as one was a CD patient, while the other was healthy and no genetic test could differentiate them. Additionally, the CD-test individual CD_47 and CD-train individual S045 were found to be monozygotic twins or, more likely, duplicate samples (kinship coefficient of 0.4743) sequenced in two different batches; we therefore removed CD_47 from CD-test as well. Thus, 62 individuals from CD-test were used for AVA,Dx performance evaluation.
3. In the WTCCC panel, 22 pairs of individuals were shown to be duplicates with kinship coefficients of ~0.5. We removed one individual from each pair, retaining 2,488 non-duplicated individuals in the WTCCC panel.
4. There were no related individuals within the GTEx panel.

Section 4 - Comparison of different gene scoring schemes

We obtained a list of Crohn's disease-related genes as annotated by the Pascal algorithm (6) from the Crohn's GWAS summary statistics (*PascalGWAS* set as described in Methods). Genes were mapped to Uniprot protein entries for use of the AVA,Dx pipeline. In addition to the *gene_score* for each gene (Methods), we also calculated other score versions:

$$gene\_score(g) = \sum_{i}^{N_g} het_i \times v\_score_i \qquad \textbf{(Eqn. 1)}$$

(1) easy_*gene_score*, as in Eqn. 1, but non-synonymous v_score=1
(2) binary_*gene_score*, as in Eqn. 1, but SNAP-neutral non-synonymous v_score=0.055 and effect non-synonymous v_score=1
(3) *EV_gene_score*, equal to the number of exonic variants in the gene
(4) *NSV_gene_score*, equal to the number of nonsynonymous variants in the gene

We used these different schemes to assign scores to *PascalGWAS* gene and trained models in *leave-one-out* cross-validation using the CD-train panel. All scoring schemes performed better than random (Supplementary Figure 1), but *gene_score* and *NSV_gene_score* outperformed others. Note that the *EV_gene_score* had the worst performance, indicating that a simple exonic variant count per-gene could not

represent the *PascalGWAS* gene signals efficiently. The best performing *NSV_gene_score*-ing model contained 75 genes and reached a PR curve AUC of 0.739 ± 0.010 (mean ± SD) and a corresponding ROC AUC of 0.665 ± 0.010. *Gene_score*-based model contained 175 genes and attained PR AUC = 0.728 ± 0.007 and ROC AUC = 0.702 ± 0.008 (Supplementary Figure 1). Since the CD-train data was not significantly skewed in the class distribution (64 CDs *vs.* 47 HCs), ROC AUC is a valid measurement of performance (7). Here, the *gene_score*-ing model achieved a higher ROC AUC while having comparable PR AUC.

In further testing, feature selection (FS) and *leave-one-out* cross-validation were done exactly as described in Methods using both *gene_score* and *NSV_gene_score*-ing schemes. Here, *NSV_gene_score* with *DKMcost* or *KS5* FS performed much worse than *gene_score*-based models, suggesting all further use of *gene_score*-ing.

Section 5 - Known CD genes are poorly informative of individual health status

We performed *leave-one-out* cross-validation training with several external gene sets: (1) literature-annotated CD genes (*MeSH set*); (2) genes mapping to known GWAS-established CD loci (*unranked-GWAS set*); (3) Swiss-Prot annotated CD genes (*SP set*); and (4) genes reported to be VEO IBD-associated by Uhlig. *et al.* (*VEO set*); (5) all genes where at least one individual in CD-train had at least one variant (*ALL set*).

To extract the *MeSH set*, for each human protein entry in Swiss-Prot (20,204 entries, September 13th, 2015) set we collected all gene and protein names, mapped to the protein entry identifier. All single word names longer than two characters were included into the search. The Swiss-Prot identifier, minus the "_HUMAN" suffix was also included as a name. Names were normalized as follows:

1. Multi-word names were converted into bags-of-words. The abstracts were searched for a combination of all words.
2. In a multi-word name, the word separators were any combination of space " ", parenthesis "(", ")", colon ":", or semicolon ";".
3. All single words were normalized by replacing any dash "-", forward slash "/", star "*", comma ",", carrot "^", and period "." with a star "*" in the middle of a word and by removing them at the ends.

All PubMed abstracts were normalized in the same fashion as names. We searched these abstracts for the occurrence of names in CD publications ("crohn disease" MeSH

term, *MeSH set*, 2,471 genes, computed on October 27th, 2015; 1,824 genes from the *MeSH set* were represented in the CD-train individuals).

To create the *GWAS set*, we extracted a set of all 1,286 validated protein-coding genes in the 163 known CD-associated regions (*GWAS set*, 925 genes from *GWAS set* were in CD-train) (8).

Since there is no ranking of the importance of genes in any of these set, we used different numbers of randomly selected genes from each set for the cross-validation, running the selection 1,000 times for each gene number/set combination. In text and figures, a subscript letter *r* before the gene number means a random selection of genes from this set instead of top ranked genes (e.g. $GWAS_{r100}$ indicates 100 genes randomly selected from the *GWAS set*).

As expected, models that were built on the *ALL set* genes were random in performance with any of the gene scoring scheme (Supplementary Figure 2D). Increasing the number of genes per subset used for model building did not improve performance. Interestingly, *GWAS* and *MeSH* sets had similar results as the *ALL* set (Supplementary Figure 2A and 2B). The best performance was achieved by using all 36 *VEO* genes *gene_score* (ROC AUC = 0.578 and area under precision-recall curve, PR AUC = 0.624). While disappointing, the inability of these models to differentiate healthy individuals from disease affected ones, suggests that there is no sequencing or scoring artifact that could differentiate CDs and HCs in the CD-train panel.

Section 6 - Performance of the GWAS-based models

We evaluated the predictions of (1) the previously reported logistic regression model based on 573 CD loci (9) and (2) the PRS method based on 230 EUR CD-associatd loci from Liu *et al.*. This logistic regression model was developed using from SNP arrays of over 13,000 individuals with CD-associated loci extracted via L1 regularization. For each of our panels, we used all available loci from this set. Specifically, only 31 SNPs were covered by the CD-train panel, 32 by CD-test panel, 480 by WTCCC panel, and 496 by the GETx panel. For the missing SNPs, we removed the corresponding coefficients from the equation. While this approach could decrease the prediction power of the model, there was no other way to resolve this problem. Note that this situation also illustrates the overarching problem of making predictions for new data sets on the basis of previously established genetic markers (see Batch effect section below for more details). As expected, the logistic regression

model performance was better than random for all panels (Supplementary Table 4). For the CD-test panel it was on par with AVA,Dx. However, for both CD-train and WTCCC-GTEx panels, AVA,Dx achieved much better performance (Supplementary Table 4 and 5) in terms of ROC/PR AUC and MCC. For the PRS method, CD-train and CD-test panels both covered 22 loci; WTCCC-GTEx panel coverd all 230 loci. Similarly as the logistic regression method, we simply removed the un-covered loci from the PRS equation for risk score calculation. The PRS method also performed better than random for all panels (Supplementary Table 6). But AVA,Dx still achieved better performance in all panels, especially in the large WTCCC-GTEx panel.

Section 7 - Removing batch effect across panels

Different sequencing platforms and variant call algorithms impose batch effects on genetic data. Generally, a prediction model is built using one batch of data, here CD-train in our study. New sequencing data often does not cover exactly the same loci as the training data (Supplementary Table 2 and 3). Thus, for a generic risk model that uses specific variants, the only solution is to impute the missing loci, which may introduce spurious noise, and to ignore the new loci, which may decrease the model predictive power. Moreover, different types of sequencing/sequence-analysis may introduce variant determination bias even for the shared loci.

By default, AVA,Dx uses the variants of the CD-train panel that cover, in total, 83,879 exonic loci. Our evaluation sets include both whole exome sequencing and whole genome sequencing data from different platforms, each covering >50% of the CD-train loci. In computing our gene scores, we inherently assume that the missing variants may be homozygous reference in additional sets – an assumption that may be false, but not expected to be crucial for our model. In this iteration of AVA,Dx we also ignored the new (not present in CD-train) variants. However, our testing (data not shown) suggests that normalizing gene scores to account for the number of the variants present may allow us to include these in the future. For all other types of differences, we were able to remove batch effects using a statistical approach used in batch effect removal in expression data – ComBat (10). Our results suggest that this method helps generate sufficiently accurate predictions. However, for more accurate predictions going forward, new individual genomes should be sequenced using the exact same protocols as the training set.

Section 8 - AVA,Dx performance for non-Europeans

All samples of the CD-train panel were collected in Kiel, Germany. The genetic information confirmed that all individuals were European (GBR or CEU, data not shown). In the Results section, we reported AVA,Dx performance on all Europeans (EUR) from CD-test (all EUR), WTCCC (94% EUR), and GTEx (86% EUR) panels. Although GWAS studies from different populations yield different variants of significance, AVA,Dx uses per-gene functional deficiency scores which focuses more on the protein function changes of the disease genes. We thus believe that the predictor could also be used in ethnically different populations. Here, we report AVA,Dx performance on non-European individuals from WTCCC (168 non-Europeans) and GTEx (91 non-Europeans) panels (Supplementary Data 2). For these 259 individuals, we reached a performance of ROC/PR AUC of 0.78/0.89 and an MCC of 0.473 at the default cutoff (Supplementary Table 5). A larger set of people is needed for further validation, but this performance suggests that AVA,Dx can indeed be applied to infer CD predisposition across ethnicities.

Section 9 - Association tests fail to identify CD-associated SNVs

We applied association tests (PLINK (11)) to the CD-train panel. Specifically, 2-sided Fisher's Exact test with Benjamini-Hochberg correction (12) was used to asses the differences in the CD vs. HC allelic counts of each variant. Two variants were found to be significantly associated with the phenotypes (rs782662688 and rs3088113, corrected p-val = 0.03 and 0.04, in coding regions of SHANK2 and CKAP4 genes, respectively).

Variant rs782662688 (Supplementary Table 1; reference A, variant T) was heterozygous in 17 HCs and homozygous reference in all CDs of the CD-train panel (allele frequency, AF_T = 0.181 in HCs and AF_T = 0 in CDs). There were 103 HC individuals in WTCCC that were heterozygous for this variant (AF_T = 0.0194, all CD), while GTEx sequencing did not cover this variant.

Both heterozygous and homozygous alternatives of the triallelic (in CD-train only) rs3088113 (Supplementary Table 1; reference C, variants T and G) existed in both CD and HC individuals in CD-train, but variant AF in HCs (AF_{TandG} = 0.287) was higher than in CDs (AF_{TandG} = 0.164). AF of (the only one existing) version of this variant in the CD-test panel was similar for CDs and HCs ($AF_{alleleT}$ = 0.092 and $AF_{alleleT}$ = 0.077, in CDs and HCs, respectively). The GTEx panel contained 144 heterozygous and 9

homozygous individuals (AF_T = 0.128), while WTCCC sequencing did not cover this variant.

Thus, these significance of associations of these two variants was not confirmed in CD-test, WTCCC, or GTEx panels. Furthermore, the allele balance (13) calculations for the two variants in the CD-train panel suggested that some of the heterozygous genotypes could be false positive calls regardless of high genotype quality. To confirm the true phenotype associations of these two variants, further validations are needed.

SUPPLEMENTARY FIGURES

Supplementary Figure 1

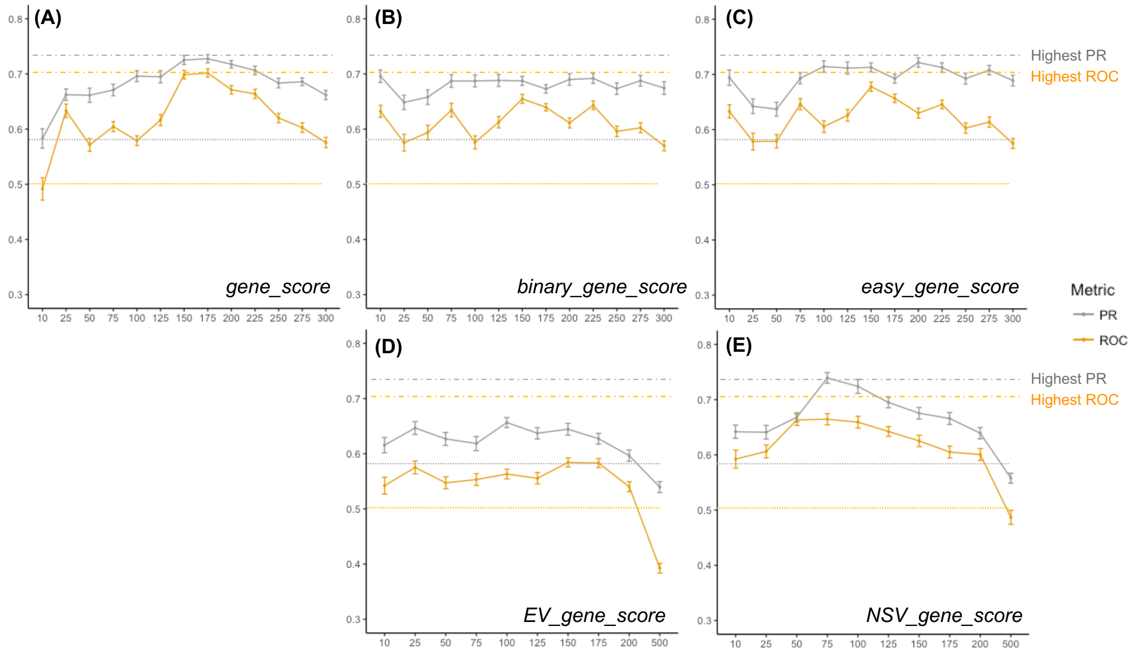

Supplementary Figure 1. Evaluating gene scoring schemes by model performance on CD-train data in *leave-one-out* cross-validation with *PascalGWAS* genes. Scoring schemes as described in Supplementary Method section 5: (**A**) gene_score, (**B**) binary_gene_score, (**C**) easy_gene_score, (**D**) EV_gene_score (exonic variant count per gene), and (**E**) NSV_gene_score (non-synonymous variant count per gene). In all plots, x-axis is the number of genes used; y-axis is AUC of PR (grey) and ROC (yellow) curves. Highest PR and ROC lines represent the peak of the curves across all schemes: here, highest PR AUC is attained by *NSV_gene_score* at 75 genes and highest ROC AUC by *gene_score* at 175 genes.

Supplementary Figure 2

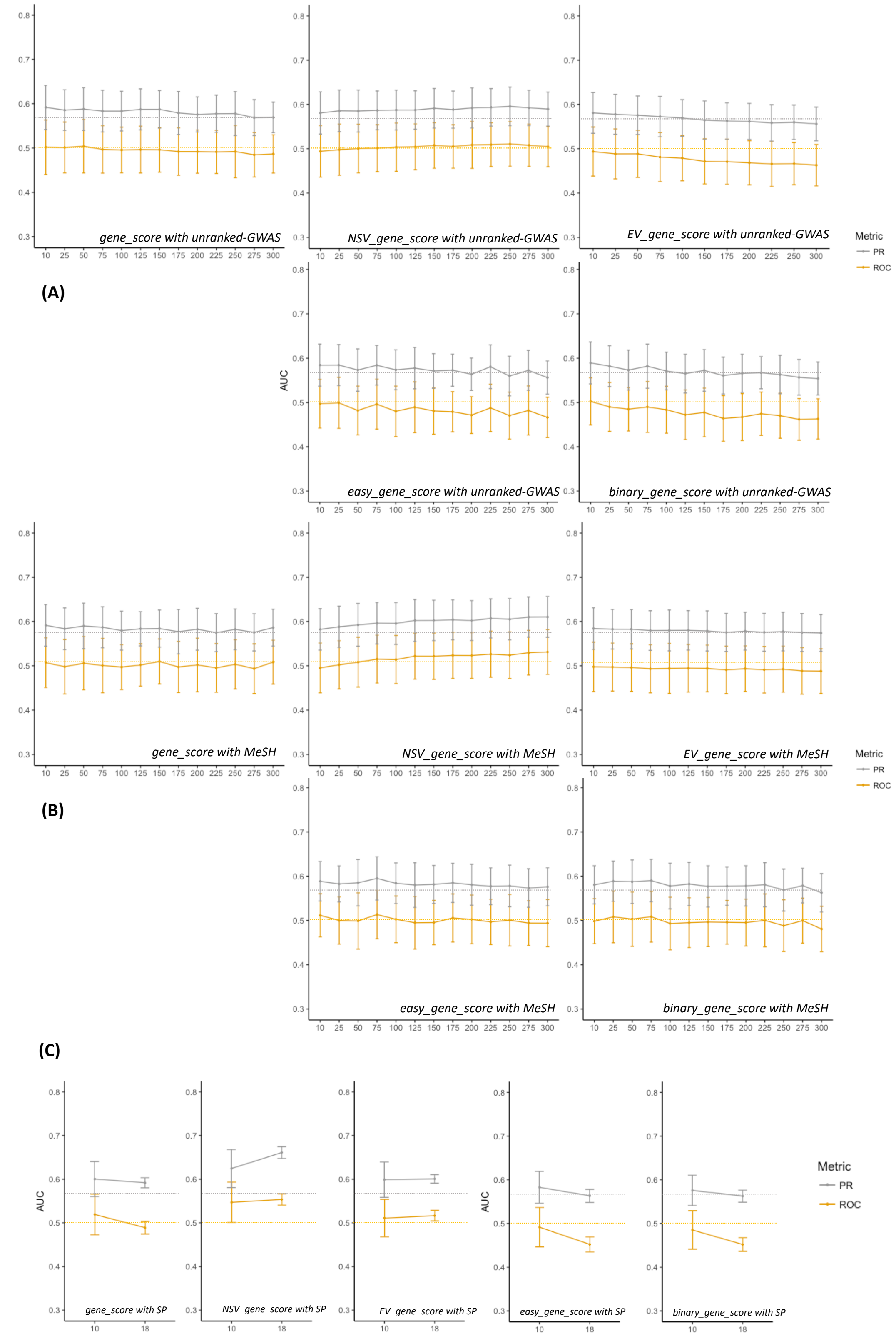

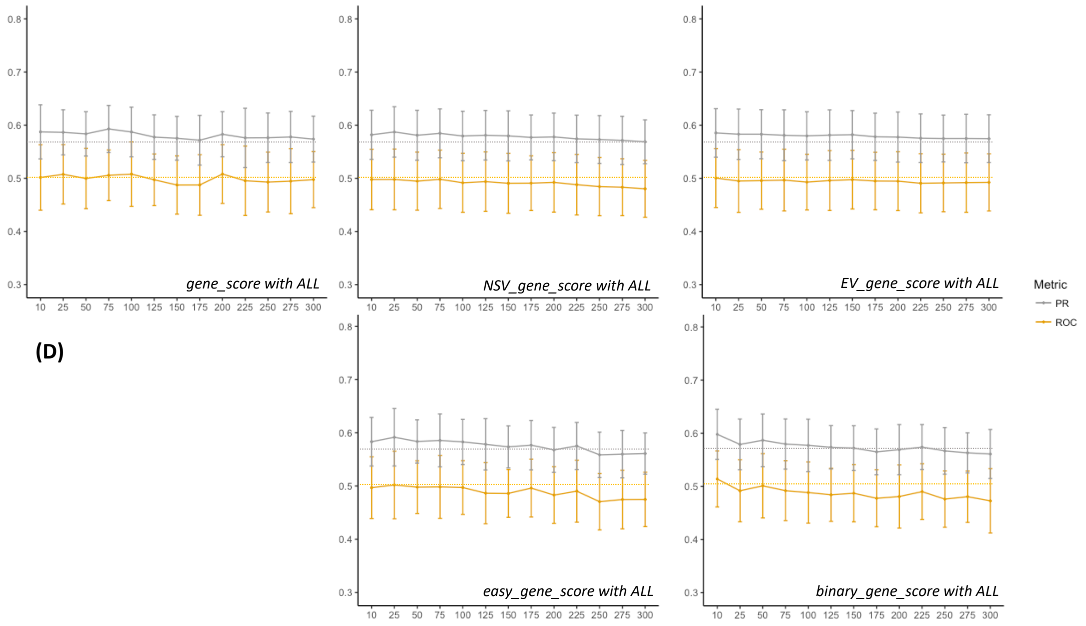

Supplementary Figure 2. Model performance on CD-train data in *leave-one-out* cross-validation across different gene sets and scoring schemes. Gene sets and scoring schemes as described in Supplementary Method sections 5 and 6 (A) unranked GWAS genes, (B) MeSH genes, (C) SP (Swiss-Prot) genes, and (D) ALL genes with different gene scoring schemes. In all plots, x-axis is the number of genes used; y-axis is AUC of PR (grey) and ROC (yellow) curves.

Supplementary Figure 3

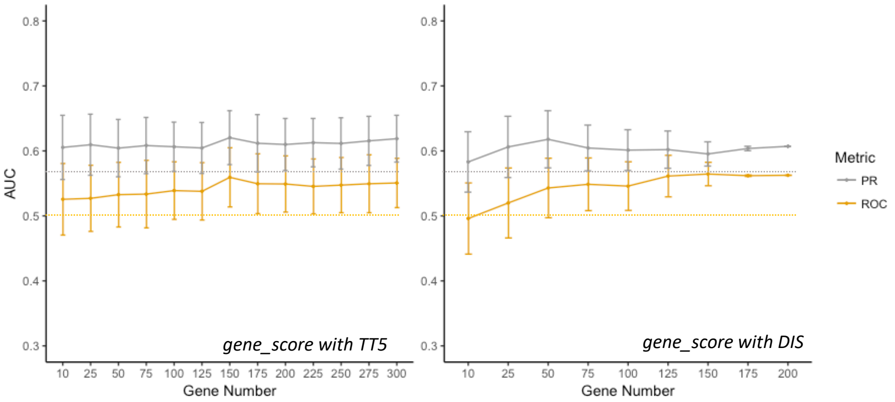

Supplementary Figure 3. Model performance on CD-train data in *leave-one-out* cross-validation on TT5 and DIS set genes. In all plots, x-axis is the number of genes used; y-axis is AUC of PR (grey) and ROC (yellow) curves.

Supplementary Figure 4

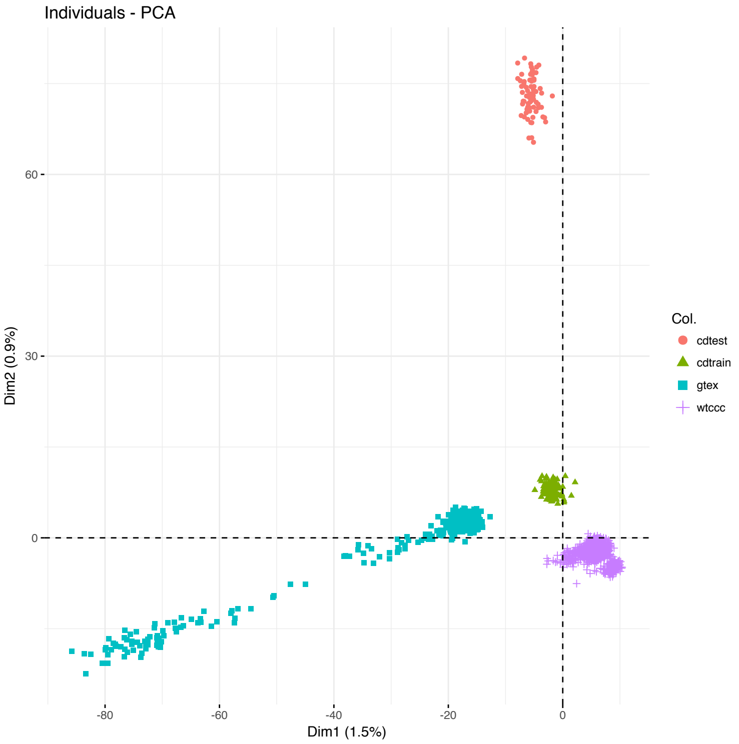

Supplementary Figure 4. Principal component analysis of four panels used in this study.

Supplementary Figure 5

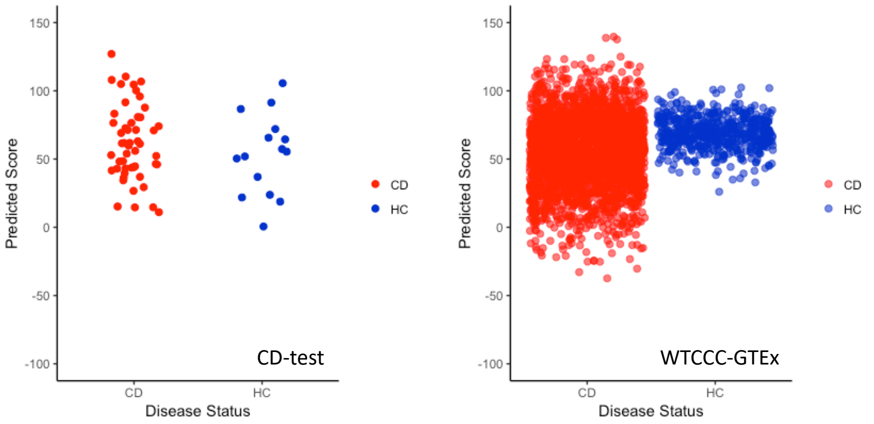

Supplementary Figure 5. Prediction of CD-test and WTCCC-GTEx individuals with AVA,Dx using *PascalGWAS*$_{175}$ genes. In the WTCCC-GTEx data, WTCCC individuals are CD and GTEx individuals are HC.

SUPPLEMENTARY TABLES

Supplementary Table 1 – Details of association analysis

Variant	REF	ALT	gnomAD*	GT	CD-train[^]		CD-train CD	CD-train HC	CD-test[^]		CD-test CD	CD-test HC	p-val[$]	WTCCC & GTEx[^]		WTCCC CD	GTEx HC	p-val[$]
					CD (64)	HC (47)			CD (49)	HC (13)				CD (2656)	HC (635)			
rs782662688<br>(in gene *SHANK2*)	A	T	$AF_{(T)}$ = 0.000053	AA	64	30	$AF_{(T)}$ = 0	$AF_{(T)}$ = 0.181	49	12	$AF_{(T)}$ = 0	$AF_{(T)}$ = 0.038	0.2097	2553	NA	$AF_{(T)}$ = 0.0194	NA	NA
				AT	0	17			0	1				103	NA		NA	
				TT	0	0			0	0				0	NA		NA	
rs3088113<br>(in gene *CKAP4*)	C	T/G	$AF_{(T)}$ = 0.1374<br>$AF_{(G)}$ = 0.03523	CC	45	21	$AF_{(T)}$ = 0.164<br>$AF_{(G)}$ = 0	$AF_{(T)}$ = 0.117<br>$AF_{(G)}$ = 0.170	40	11	$AF_{(T)}$ = 0.092<br>$AF_{(G)}$ = 0	$AF_{(T)}$ = 0.077<br>$AF_{(G)}$ = 0	1 (for T)<br>1 (for G)	NA	480	NA	$AF_{(T)}$ = 0.128<br>$AF_{(G)}$ = 0	NA
				CT	17	9			9	2				NA	144	NA		
				TT	2	1			0	0				NA	9	NA		
				CG	0	16			0	0				NA	0	NA		
				GG	0	0			0	0				NA	0	NA		

* Allele frequencies from gnomAD are for European (non-Finnish) population
^ Numbers of individuals are in parenthesis
$ p-value was calculated by two-sided Fisher's Exact Test

Supplementary Table 2 – Exonic variant locus overlap across the four sets

	**CD-train**	**CD-test**	**WTCCC**	**GTEx**
**CD-train**	83,879	44,999 (53.6%)	69,664 (83.1%)	65,735 (78.4%)

Supplementary Table 3 – Variant locus overlap in $DKMc_{125}$ genes in the four sets

	**CD-train**	**CD-test**	**WTCCC**	**GTEx**
**CD-train**	1,238	723 (58.4%)	1,050 (84.8%)	1,013 (81.8%)

Supplementary Table 4 - Performance of the 573-locus logistic regression model

Panels	ROC AUC	PR AUC^	TP$	FP$	TN$	FN$	Precision	Recall	NPV	MCC	F1 for CD
CD-train	0.63	0.74	46	26	21	18	64%	72%	54%	0.17	0.676
CD-test	0.73	0.92	16	1	12	33	94%	33%	27%	0.23	0.485
WTCCC and GTEx*	0.59	0.85	2476	545	90	180	82%	93%	33%	0.11	0.872

* As the regression model was built on a mixed population, all individuals from WTCCC and GTEx including EURs and non-EURs were evaluated.
^ Baseline PR AUCs for CD-train, CD-test, and WTCCC-GTEx panels are: 0.58, 0.79, 0.81
$ TP, FP, TN, and FN were calculated using the default cutoff 0.5 suggested in the paper describing the model

Supplementary Table 5 – Detailed Performance of AVA,Dx

Panels	ROC	PR^ (CD)	PR^ (HC)	Cutoff$	TP$	FP$	TN$	FN$	Precision	Recall	NPV	MCC	F1 for CD	F1 for HC
CD-train*	0.75	0.80	0.69	45	17	1	46	47	94.4%	26.6%	49.5%	0.328	0.415	0.657
				14.3	47	19	28	17	71.2%	73.4%	62.2%	0.332	0.723	0.609
				0	57	25	22	7	69.5%	89.1%	75.9%	0.403	0.781	0.579
CD-test	0.69	0.92	0.35	45	9	0	13	40	100.0%	18.4%	24.5%	0.212	0.310	0.394
				14.3	36	8	5	13	81.8%	73.5%	27.8%	0.107	0.774	0.323
				0	44	11	2	5	80.0%	89.8%	28.6%	0.067	0.846	0.200
WTCCC and GTEx (EUR)	0.76	0.94	0.31	45	384	1	543	2104	99.7%	15.4%	20.5%	0.176	0.267	0.340
				14.3	1432	68	476	1056	95.5%	57.6%	31.1%	0.346	0.718	0.459
				0	1924	242	302	564	88.8%	77.3%	34.9%	0.279	0.827	0.428
WTCCC and GTEx (non-EUR)	0.78	0.89	0.55	45	31	0	91	137	100%	18%	39.9%	0.271	0.312	0.571
				14.3	107	13	78	61	89%	64%	56.1%	0.473	0.743	0.678
				0	137	50	41	31	73%	82%	56.9%	0.283	0.772	0.503
WTCCC and GTEx (total)	0.76	0.94	0.33	45	415	1	634	2241	99.8%	15.6%	22.1%	0.184	0.270	0.361
				14.3	1539	81	554	1117	95.0%	57.9%	33.2%	0.357	0.720	0.480
				0	2061	292	343	595	87.6%	77.6%	36.6%	0.276	0.823	0.436

* CD-train performance was from one of 1,000 re-samplings

^ Baseline PR AUCs for CD-train, CD-test, WTCCC-GTEx (EUR), WTCCC-GTEx (non-EUR), and WTCCC-GTEx (total) panels are: 0.58, 0.79, 0.82, 0.65, and 0.81, respectively, for CD; 0.42, 0.21, 0.18, 0.35, and 0.19, respectively, for HC.

$ We suggest three different cutoffs: optimum CD precision (45), best separation of two classes (14.3), and optimum CD recall (0).

Supplementary Table 6 - Performance of the PRS (Polygenic Risk Score) method

Panels	ROC	PR* (CD)	PR* (HC)	TP^	FP^	TN^	FN^	Precision	Recall	NPV	MCC	F1 for CD	F1 for HC
CD-train	0.65	0.74	0.54	58	34	13	6	63%	91%	68%	0.24	0.744	0.394
CD-test	0.70	0.90	0.38	47	11	2	2	81%	96%	50%	0.19	0.879	0.235
WTCCC and GTEx	0.57	0.84	0.24	1015	192	443	1663	84%	38%	21%	0.06	0.523	0.323

* Baseline PR AUCs for CD-train, CD-test, and WTCCC-GTEx (total) panels are: 0.58, 0.79, and 0.81, respectively, for CD; 0.42, 0.21, and 0.19, respectively, for HC.

^ Default cutoff is zero for GRS —no risk allele

SUPPLEMENTARY REFERENCES

1. McKenna, A., Hanna, M., Banks, E., Sivachenko, A., Cibulskis, K., Kernytsky, A., Garimella, K., Altshuler, D., Gabriel, S., Daly, M. *et al.* (2010) The Genome Analysis Toolkit: A MapReduce framework for analyzing next-generation DNA sequencing data. *Genome Res*, **20**, 1297-1303.
2. Pattnaik, S., Vaidyanathan, S., Pooja, D.G., Deepak, S. and Panda, B. (2012) Customisation of the Exome Data Analysis Pipeline Using a Combinatorial Approach. *Plos One*, **7**.
3. Altshuler, D.M., Durbin, R.M., Abecasis, G.R., Bentley, D.R., Chakravarti, A., Clark, A.G., Donnelly, P., Eichler, E.E., Flicek, P., Gabriel, S.B. *et al.* (2012) An integrated map of genetic variation from 1,092 human genomes. *Nature*, **491**, 56-65.
4. Zheng, X.W., Levine, D., Shen, J., Gogarten, S.M., Laurie, C. and Weir, B.S. (2012) A high-performance computing toolset for relatedness and principal component analysis of SNP data. *Bioinformatics*, **28**, 3326-3328.
5. Manichaikul, A., Mychaleckyj, J.C., Rich, S.S., Daly, K., Sale, M. and Chen, W.M. (2010) Robust relationship inference in genome-wide association studies. *Bioinformatics*, **26**, 2867-2873.
6. Lamparter, D., Marbach, D., Rueedi, R., Kutalik, Z. and Bergmann, S. (2016) Fast and Rigorous Computation of Gene and Pathway Scores from SNP-Based Summary Statistics. *PLoS Comput Biol*, **12**, e1004714.
7. Davis, J. and Goadrich, M. (2006), *Proceedings of the 23rd international conference on Machine learning*. ACM, pp. 233-240.
8. Jostins, L., Ripke, S., Weersma, R.K., Duerr, R.H., McGovern, D.P., Hui, K.Y., Lee, J.C., Schumm, L.P., Sharma, Y., Anderson, C.A. *et al.* (2012) Host-microbe interactions have shaped the genetic architecture of inflammatory bowel disease. *Nature*, **491**, 119-124.
9. Wei, Z., Wang, W., Bradfield, J., Li, J., Cardinale, C., Frackelton, E., Kim, C., Mentch, F., Van Steen, K., Visscher, P.M. *et al.* (2013) Large Sample Size, Wide Variant Spectrum, and Advanced Machine-Learning Technique Boost Risk Prediction for Inflammatory Bowel Disease. *Am J Hum Genet*, **92**, 1008-1012.
10. Johnson, W.E., Li, C. and Rabinovic, A. (2007) Adjusting batch effects in microarray expression data using empirical Bayes methods. *Biostatistics*, **8**, 118-127.
11. Purcell, S., Neale, B., Todd-Brown, K., Thomas, L., Ferreira, M.A.R., Bender, D., Maller, J., Sklar, P., de Bakker, P.I.W., Daly, M.J. *et al.* (2007) PLINK: A tool set for whole-genome association and population-based linkage analyses. *Am J Hum Genet*, **81**, 559-575.
12. Benjamini, Y. and Hochberg, Y. (1995) Controlling the False Discovery Rate - a Practical and Powerful Approach to Multiple Testing. *J Roy Stat Soc B Met*, **57**, 289-300.
13. Li, H. (2014) Toward better understanding of artifacts in variant calling from high-coverage samples. *Bioinformatics*, **30**, 2843-2851.
